# Investigating socioecological obesogenic factors in children with Autism Spectrum Disorder

**DOI:** 10.3389/fpubh.2022.867456

**Published:** 2022-10-06

**Authors:** TaeEung Kim, Eun Hye Kwon

**Affiliations:** ^1^Department of Preventive Medicine, School of Medicine, Kyung Hee University, Seoul, South Korea; ^2^Department of Counseling, Health & Kinesiology, Texas A&M University-San Antonio, San Antonio, TX, United States

**Keywords:** Autism Spectrum Disorder, obesity, children, wellbeing, socioecological factors

## Abstract

Obesity-related information in children with Autism Spectrum Disorder (ASD) is limited, and research findings are contradictory. Thus, this study aimed to use a nationwide non-clinical sample to examine the association of sociological factors with overweight status in children with ASD and reveal the degree of differences in the risk factors for overweight in children with and without ASD. The data for this cross-sectional study, based on the modified ecological system theory model, were obtained from the 2019 National Survey of Children's Health. The weighted logistic regressions were performed to determine the factors associated with overweight status in children with ASD, controlling for demographics, physical activity-related behaviors, and family and environmental conditions. A total of 529 children were identified (mean age 13.78 years, 83.21% boys). Two-parent households, less healthy parents and households, households with smokers, poor sleep quality, and greater participation in organized activities were associated with a higher likelihood of overweight in children with ASD (all *P* < 0.05). The determinants of obesity among children with ASD go beyond the individual level; family and community support are important. Therefore, greater attention should be directed toward the families of children with ASD and community-level administrative policies to improve quality of life by preventing or reducing obesity in children with ASD.

## Introduction

The prevalence of childhood obesity has been well investigated. In the United States of America (US), more than one in three children and adolescents (“children” hereafter) are overweight or obese (“overweight” hereafter) (1). In childhood, considering age and sex, overweight is defined as a body mass index (BMI) above the 85th percentile but below the 95th percentile, whereas obesity is defined as a BMI above the 95th percentile (2). During the past three decades, childhood obesity in the US has quadrupled (3).

Childhood obesity negatively impacts overall health and wellbeing. Studies show that overweight children are at a higher risk of cardiovascular diseases, including high blood pressure and/or high cholesterol, (4) pre-diabetes resulting in diabetes later in life, (5) and bone and joint problems, as well as sleep problems (6). Additionally, they are at an increased risk of psychosocial problems, such as low self-esteem and stigmatization (7). Moreover, childhood obesity increases the likelihood of health problems later in life; these include obesity, (8) heart disease, type 2 diabetes, stroke, and osteoarthritis (6). There is also a high association with different types of cancer, such as breast, colon, kidney, pancreatic, thyroid, ovarian, cervical, and prostate, as well as multiple myeloma and Hodgkin's lymphoma (9).

In general, childhood obesity is strongly associated with lifestyle habits, such as a high calorie intake and inadequate physical activity (6). Such lifestyle patterns are substantially attributed to families, (10) schools, (11) communities, (12), and environments (13). Since family, school, and the community play an important role in preventing and minimizing obesity in children, it is critical to establish healthy and positive environments through appropriate policies and initiatives. In this manner, children can learn ‘about and practice healthy eating and become more physically active (14).

Several studies have sought to identify the determinants of childhood obesity. However, such research is limited in the context of Autism Spectrum Disorder (ASD), perhaps owing to the relatively few cases compared to the general population. Since children with ASD often face substantial social and behavioral challenges at both the familial and societal levels, (15) they may be more vulnerable to obesity and its associated complex behavioral, psychosocial, and physical difficulties (16). Therefore, children with ASD face extreme hardships related to learning, thinking, and problem solving (17).

Obesity in children with ASD is a complex phenomenon. The features and possible outcomes of some of the symptoms of ASD, such as lack of autonomy, self-motivation, and social inspiration, can be considered major risk factors for obesity to obesity. Additionally, children with ASD are less likely than their typically developing counterparts to participate in structured physical activity (18) or family meals, resulting in unhealthy weight (19). They are also likely to experience poor sleep quality, which can contribute to obesity (20). Limited community resources play an important role in maintaining a healthy weight in children with ASD, including parents' health, neighborhood and school safety, proper physical activity programs, and accessibility to facilities.

Studies on physical activity and sedentary behavior associated with obesity in typically developing children or at the medical level have suggested directions for the prevention and improvement of obesity in children with ASD (21–23). However, while these studies may provide insight into obesity prevention among children with ASD on a broad level, there is a limitation to understanding the related socioecological factors and prevention strategies for obesity in this population. Therefore, it is imperative to measure the level of physical inactivity, sedentary lifestyles, and community resources and to understand the correlates of overweight in children with ASD. Unfortunately, a lack of understanding of the factors associated with childhood obesity in this population diminishes the quality of public health and results in societal financial burdens (24, 25). To gain a holistic understanding of obesity and its related factors, it is crucial to draw insights from a large sample of children with ASD in a non-clinical setting.

In this context, this study aimed to examine overweight and its associated factors in a large non-clinical sample of children with ASD. It is hypothesized that in children with ASD: (1) social interactions (e.g., social involvement, voluntary activities, and family meals) are negatively associated with overweight status; (2) parents' physical and mental health status is associated with overweight status; (3) insufficient sleep is positively associated with overweight status; and (4) neighborhood conditions (e.g., accessibility of and number of parks and recreational facilities) are associated with overweight status.

## Materials and methods

### Study population and sampling

This study is a secondary analysis of the 2019 National Survey of Children's Health, (26) which provides a wealth of data on several intersecting aspects: physical and mental health; access to quality healthcare; and the child's familial, neighborhood, school, and social context. The National Survey of Children's Health is funded and administered by the Health Resources and Services Administration Maternal and Child Health Bureau. In 2019, the Census Bureau conducted a revised version of the survey *via* post and the internet. The original data pertained to 29,433 children, but as the BMI of children aged 0–9 years could not be determined, in this study, the data of 529 children aged 10–17 years, obtained from their parents, were analyzed.

**Figure 1:**
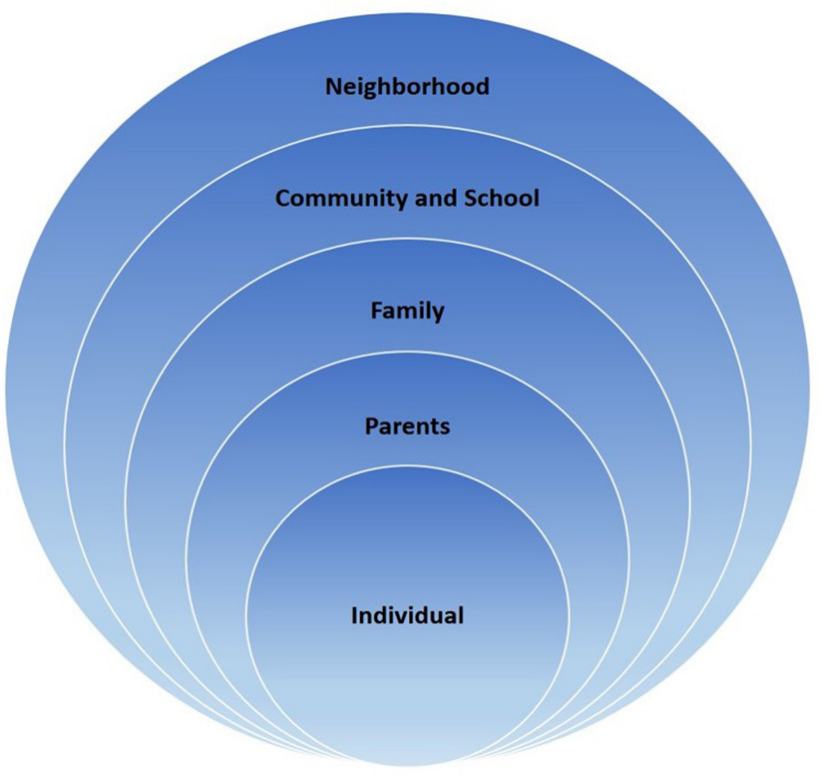
Modified socioecological theory framework from Bronfenbrenner (27).

To make it easier to understand the complex causes of obesity in children with ASD, a modified socioecological approach was employed (27). The five main parts of the framework were as follows: (1) children's age, sex, race/ethnicity, physical activity, and sedentary lifestyle; (2) parenting capacity, which included educational level, income level, and household structure; (3) family health and activities, which included parental health, drinking and smoking status, family eating patterns, and stability of food provision; (4) community and school activities, such as the existence of afterschool programs, community service, and parents' children's activities due to factors in the community and schools; and (5) neighborhood factors, such as the degree of safety and support in the neighborhood, stability of the school and neighborhood, cooperation within the neighborhood, and facilities related to children's activities in the neighborhood (Figure 1).

### Measurement

#### Overweight status

The main outcome variable was the binary variable of being overweight (≥85th BMI percentile) vs. non-overweight (0th <BMI percentile <85th) for age and sex (2). BMI was obtained by dividing weight in kilograms by the square of height in meters (28). BMI was calculated in percentiles to determine the severity of obesity using a growth chart considering the child's age and sex (29).

#### ASD

Autism Spectrum Disorder was measured as a dichotomous variable, which is whether or not the children currently had Asperger's syndrome, pervasive developmental disorders, or ASD. Obesity-related variables were extracted from five main areas: individual factors, parenting capacity, family activities, and community and school activities, neighborhood support.

#### Socioecological factors

First, among the socioecological factors related to obesity in children, individual factors included age, sex, race/ethnicity, physical activity, sedentary lifestyle, and quality of sleep. Owing to the limitations of BMI information, the age of the participants ranged from 10 to 17 years. Participants' race/ethnicity was classified into five groups: Hispanic, non-Hispanic white, non-Hispanic black, non-Hispanic Asian, and others. The level of physical activity was measured on a four-point Likert scale (1 = 0 day to 4 = every day) based on the number of days of physical activity, exercise, or sports that children participated in for at least 60 min during the past week. Sedentary lifestyle was measured on a five-point Likert scale (1 = <1 h to 5 = more than 4 h) over the past week, such as watching television; using computers, cell phones, or other electronic devices; playing online games; surfing the internet; or engaging in non-academic social networks. Sleep quality was measured as the number of hours of sleep on an average day on most weekdays during the past week and converted into a binary variable according to the American Academy of Pediatrics' sleep duration recommendations for 24-h periods (i.e., 9–12 and 8–10 h for children and adolescents aged 6–12 and 13–18 years, respectively) (30).

Childhood obesogenic factors related to parenting capacity were educational level, income level, and family structure. Parents' educational level was measured using a four-point Likert scale (1 = less than high school to 4 = college degree or higher). Parents' income level was measured on a four-point Likert scale (1 = 0–99% of the federal poverty level to 4 = 400% or greater of the federal poverty level). Family structure was initially measured on a five-point Likert scale and then restructured into binary variables (e.g., parents vs. other family structures, such as single parent and guardians).

Factors related to family health and activities contributing to children's obesity included parents' health, drinking, drug use status, stress coping ability, family meals, and food security. Parents' overall health, encompassing physical and mental health, was assessed on a four-point Likert scale. The primary caregiver received a score between 1 and 12 points, with a higher score indicating better overall health. A binary variable was used to determine whether or not a child lived with a person with alcohol or drug-related problems. The smoking status of any person living in a child's home was determined in relation to the use of cigarettes and pipe tobacco. The stress-coping variable, used to determine the frequency of instances of the household demonstrating resilience, was assessed on a three-point Likert scale (0 = 0–1 items of all or most of the time to 2 = all or most of the time to 3= all four items). The aspect of meals assessed how many days over the past week all family members had eaten together; this was done on a four-point Likert scale (1 = 0 day to 4 = every day). Food security was measured on a four-point Likert scale as the child's household's ability to afford food for the past 12 months (1 = always get enough nutritious food to 4 = often not enough).

Three childhood obesity-related community- and school-activity variables were investigated. Organized afterschool activities were assessed in terms of which organized activity or class the child had participated in after school or on weekends over the past 12 months. The child indicated the family's level of engagement in community service or volunteering over the past 12 months based on their participation in such events at school, church, or in the community. Parental event/activity participation was measured on a four-point Likert scale (1 = rarely or never to 4 = always).

Four variables of neighborhood safety and support areas were assessed. School safety and safe neighborhoods, evaluated by asking parents about the extent to which they considered their child safe in school or the neighborhood, were measured on a three-point Likert scale (1 = somewhat/definitely disagree to 3 = definitely agree). A binary variable was used to assess whether the neighborhood was supportive. A neighborhood amenities variable, which concerned how many amenities, such as parks, recreation centers, sidewalks, or libraries, that the neighborhood contained, was measured on a five-point Likert scale.

### Data analyses

Pearson's χ^2^ tests and *t*-tests with weighted counts and column percentages were performed for the descriptive statistics. The weighted logistic regression analyses were performed to investigate the relationship between BMI status and quality of life in children with ASD in terms of socioecological obesity factors, including individual factors, parenting capacity, family health and activities, and community and school activities plus neighborhood safety and support. All statistical and logistic regression analyses were performed for sex, race/ethnicity, socioeconomic status, family, school, community, and other relevant characteristics using STATA version 15.1 (StataCorp LLC, College Station, TX).

### Ethical considerations

The relevant institutional review board exempted this study.

## Results

The unweighted (i.e., number of participants and mean) and weighted (i.e., standard deviation and percentage) descriptive statistics of the sample are shown in Table 1. A total of 529 children with ASD (representing 1,291,821 children at the population level) were recruited for this study. The mean age was 13.78 (SD = 2.09) years, and 83.21% of the participants were boys. Table 1 shows that the participants belonged to diverse races and ethnicities, including non-Hispanic whites (52.19%), Hispanics (27.15%), non-Hispanic blacks (14.74%), others (4.33%), and non-Hispanic Asians (1.59%).

**Table 1:** Characteristics of study participants with overweight status.

	**% (** ***n*** **), Mean (SD)**			
**Variables**	**Overweight**	**Non-Overweight**	**Overall**	***P*** **-value**
	44.30% (194)	51.40% (311)	95.71% (529)	
**Individual**				
Age	13.62 (1.72)	13.87 (2.33)	13.78 (2.09)	0.11
Sex (Male)	85.70% (164)	81.28% (253)	83.21% (438)	0.69
**Race/ethnicity**				
Non-hispanic white	43.89% (141)	60.12% (228)	52.19% (385)	0.27
Non-hispanic black	11.90% (16)	16.29% (29)	14.74% (48)	
Hispanic	38.34% (22)	17.84% (24)	27.15% (50)	
Non-hispanic Asian	1.36% (5)	1.91% (9)	1.59% (14)	
Other	4.50% (10)	3.78% (21)	4.33% (32)	
Physical activities	2.23 (0.86)	2.22 (0.91)	2.22 (0.90)	0.55
Sedentary lifestyles	3.93 (0.92)	3.80 (1.15)	3.84 (1.05)	0.32
Sleep quality^**^	47.56% (112)	73.31% (220)	59.95% (343)	<0.01
**Parenting capacity**				
**Parents' education**				
Less than high school	3.79% (3)	8.37% (3)	6.46% (8)	0.20
High school degree or GED	29.90% (31)	14.57% (31)	22.22% (68)	
Some college or technical school	37.63% (65)	28.60% (90)	32.09% (163)	
College degree or higher	28.68% (955)	48.46% (187)	39.24% (290)	
**Household income level**				
0–99% FPL	27.94% (32)	20.87% (49)	24.31% (90)	0.22
100–199% FPL	33.15% (46)	21.66% (44)	26.58% (93)	
200–399% FPL	25.41% (61)	29.12% (107)	28.31% (177)	
400% FPL or greater	13.50% (55)	28.36% (111)	20.79% (169)	
Family structure (two parents)	63.23% (121)	63.57% (224)	62.75% (357)	0.50
**Family health and activities**				
Parents' health	7.30 (3.49)	8.17 (3.92)	7.72 (3.83)	0.16
Alcohol and drugs^*^	28.02% (30)	8.62% (40)	17.80% (75)	< 0.05
Smoking	27.86% (37)	16.97% (43)	22.07% (86)	0.37
Stress coping	1.60 (0.60)	1.56 (0.59)	1.57 (0.62)	0.54
Meal together	2.97 (0.76)	2.93 (0.95)	2.95 (0.88)	0.65
**Food security**				
Often not enough	4.10% (7)	1.98% (5)	2.84% (12)	0.11
Sometimes not enough	3.17% (9)	2.41% (10)	3.10% (22)	
Never nutritious enough	45.61% (74)	27.30% (90)	36.54% (177)	
Always nutritious enough	36.08% (102)	64.36% (199)	50.51% (308)	
**Community and school activities**				
Organized afterschool activities^*^	75.81% (122)	57.30% (190)	66.21% (327)	< 0.05
Community service or volunteer work	44.07% (71)	33.05% (113)	37.08% (189)	0.23
Parental participation in child's events/activities	3.22 (0.86)	3.22 (1.04)	3.22 (0.96)	0.47
**Neighborhood safety and support**				
Safe school	2.51 (0.55)	2.51 (0.66)	2.52 (0.62)	0.20
Supportive neighborhood	29.65% (81)	42.22% (130)	35.97% (221)	0.35
Safe neighborhood	2.43 (0.70)	2.49 (0.67)	2.47 (0.70)	0.17
Neighborhood amenities	2.51 (1.11)	2.58 (1.26)	2.54 (1.21)	0.39

n = 529; weighted n = 1,291,821.

* P < 0.05,

** P < 0.01. Significance was determined using co-variate analysis. Data source: 2019 National Survey of Children's Health. GED, general equivalency diploma; FPL, federal poverty line; SD, standard deviation.

One weighted multivariate logistic analysis considering socioecological obesogenic factors was performed (i.e., personal factors, parenting capacity, family health and activities, community and school activities, and neighborhood safety and support). Five independent variables associated with children's overweight status had significantly different odds ratios (ORs) in their binary BMI levels [i.e., overweight (≥85th percentile) vs. non-overweight (0th <BMI percentile <85th percentile)] (see Table 2).

**Table 2:** Weighted multivariate logistic regression results.

**Co-variates**	**OR**	**95% CI**
**Individual**		
Age (years)	0.87	(0.75, 1.03)
Sex (Male)	0.98	(0.42, 2.25)
**Race/ethnicity:**		
Non-hispanic white	–	
Non-hispanic black	0.68	(0.20, 2.29)
Hispanic	1.67	(0.63, 4.47)
Non-hispanic Asian	3.58	(0.42, 30.51)
Other	1.49	(0.44, 5.03)
Physical activities	1.32	(0.96, 1.82)
Sedentary lifestyles	1.23	(0.89, 1.69)
Sleep quality	0.37^**^	(0.19, 0.71)
**Parenting capacity**		
**Parents' education**		
Less than high school	–	
High school degree or GED	18.70	(0.96, 362.67)
Some college or technical school	9.37	(0.49, 179.45)
College degree or higher	9.79	(0.50, 190.23)
**Household income level**		
0–99% FPL	–	
100–199% FPL	1.03	(0.31, 3.41)
200–399% FPL	0.81	(0.30, 2.21)
400% FPL or greater	0.68	(0.21, 2.20)
Family structure (two parents)	5.70^**^	(1.83, 17.79)
**Family health and activities**		
Parents' health	0.80^**^	(0.68, 0.93)
Alcohol and drugs	1.86	(0.74, 4.65)
Smoking	2.62^*^	(1.03, 6.65)
Stress coping	0.97	(0.57, 1.65)
Meal together	0.76	(0.52, 1.09)
**Food security**		
Often not enough	–	
Sometimes not enough	0.11	(0.01, 5.84)
Never nutritious enough	0.09	(0.01, 2.77)
Always nutritious enough	0.07	(0.01, 2.06)
**Community and school activities**		
Organized afterschool activities	2.74^**^	(1.35, 5.55)
Community service or volunteer work	1.37	(0.69, 2.71)
Parental participation in child's events/activities	0.88	(0.61, 1.28)
**Neighborhood safety and support**		
Safe school	1.15	(0.70, 1.89)
Supportive neighborhood	0.60	(0.26, 1.37)
Safe neighborhood	1.34	(0.75, 2.40)
Neighborhood amenities	1.30	(0.96, 1.76)

* P < 0.05,

** P < 0.01; 019 National Survey of Children's Health; Weighted N = 1,291,821; OR, odds ratio; CI, confidence interval; GED, general equivalency diploma; FPL, federal poverty level.

In terms of obesogenic factors, children who slept well had statistically significantly lower odds of being overweight than those who did not receive adequate sleep [OR = 0.38, 95% confidence interval (CI) = 0.19–0.71]. According to parenting capacity, children from two-parent households (OR = 5.70, 95% CI = 1.83–17.79) were more likely to be overweight than children from any other family structure (e.g., one parent, no parents, etc.). Regarding family health and activities, a one-unit increase in parents' health resulted in a lower risk of being overweight (OR = 0.80, 95% CI = 0.68–0.93). Children from households with smokers had 2.62 times higher odds of being overweight than children from households without smokers (OR = 2.62, 95% CI = 1.03–6.65). In terms of community and school activities, children who had participated in more organized activities after school or on weekends over the past 12 months were more likely to be overweight than those who had not (OR = 2.74, 95% CI = 1.35–5.55).

## Discussion

This cross-sectional study examined overweight children with ASD as well as obesogenic factors in this large non-clinical sample. According to the results, the risk factors for obesity in children with ASD were both similar to and different from those reported in a previous study (31). The present study revealed the complexity of the obesity trend in children with ASD in terms of socioecological, multilevel factors.

Physiologically, common comorbidities observed among individuals with ASD include neurological, psychiatric, and physical conditions (32). Neurological comorbidities in ASD, including sleep impairment, sensory abnormalities, and psychiatric conditions, include delays and/or deficits in motor function and attention deficit hyperactivity disorder (32). Some of the medications prescribed to manage the symptoms of these comorbidities have been linked to weight gain, but this aspect has not been explored in previous studies. Egan et al. (33) determined that the psychotropic medication status of children with ASD is not related to the BMI *z*-score. In summary, individuals with ASD share physiological comorbidities. However, these comorbidities and the prescribed medications cannot be considered as the only factors contributing to overweight in this population. This is the major reason why interdisciplinary analysis is more appropriate for determining obesity factors in ASD.

Previous studies indicate that children with ASD are at a greater risk of being overweight than their typically developing counterparts (33). In the past few decades, many multidisciplinary studies have been conducted to investigate the causes of childhood obesity. These have determined that the major causes of obesity, such as activity level, diet, behavior, and socioeconomic status, interact with each other to increase the obesity rate in children (34–38). We observed the same trend among children with ASD. Several factors such as the level of school activity, ethnic background, community support, and parental status interact and affect the increase in obesity rate among children with ASD. Various obesogenic factors, such as diet, physical activity, and sleep patterns within the context of the family environment have been confirmed (31); thus, family dynamics are the main criteria for any examination of childhood obesity risk factors (31).

In this study, social and community support were also observed to contribute to the development of obesity among children with ASD. Research indicates that social and community support for families of children with ASD improves family adaptation (39). Social support can improve the views of family members concerning their family structure, and optimistic viewpoints are important in the development of helpful coping strategies for eventual family adaptation (40). However, community support is not equally accessible to every family with children with ASD that needs help (41). Since there is a positive relationship between social support, community support, and obesity, systematic support needs to be established for parents of children with ASD after their child's diagnosis.

Curtin et al. (31) indicated that the challenges of engaging in physical activity might be uniquely associated with the development of obesity in children with ASD. One of the main reasons for insufficient physical activity is the limited number of school-based extracurricular physical activity programs for children with ASD (42, 43). Additionally, increased time spent engaging in sedentary behavior has been shown to increase the risk of obesity among children with ASD. Since factors that lead to sedentary behavior and lack of engagement in physical activity among children with ASD are interconnected with their social and/or behavioral impairments, they may encounter more difficulties in participating in formal and informal forms of physical activity than typically developing children. The greater risk of overweight among children with ASD was explored in a meta-analysis (44). Based on their results, ethnicity (non-Caucasian race), increasing age, and female sex are potential risk factors for overweight in children with ASD (44).

These studies clarify that it is not possible to identify a single factor as leading to overweight among children with ASD. Therefore, a socioecological approach is crucial. As hypothesized, social interactions (e.g., social involvement, voluntary activities, and family meals) and parents' physical and mental health status played a positive role, whereas insufficient sleep played a negative role. Additionally, neighborhood conditions (e.g., accessibility of and the number of parks and recreational facilities) were associated with overweight status in children with ASD.

Multiple studies have determined the relationship between environmental factors and obesity status in typically developing children, including maternal obesity, (45, 46) lower educational attainment, (47) lower physical activity rates, (48) poor nutrition knowledge, (48) food insecurity, (49) smoking, (45), and rules regarding food consumption and eating at regular times (50). In this study, national-level data from the US were analyzed to determine the relationship between the obesity status of children with ASD and environmental factors. The results indicated a trend between obesity level and sleep patterns, family structure, and household smoking status. Studies on the effect of paternal/household smoking on childhood overweight have reported inconsistent results; some studies have found no association between household smoking and childhood overweight, (51, 52) whereas others have observed positive associations (53–55).

Cross-sectional studies have reported mixed results regarding the association between sleep duration and obesity in children and adolescents. However, sleep deprivation is believed to lead to fatigue, daytime sleepiness, cognitive problems, low activity levels, and changes in the levels of several hormones (56, 57). Hormones that could be affected by sleep deprivation include leptin, ghrelin, insulin, cortisol, and growth hormone (58, 59). Hormonal changes may contribute to energy imbalance, which could lead to overweight or obesity. In this study, a similar trend was observed among children with ASD, indicating a negative correlation between sleep duration and obesity. This indicates that in children with ASD, short sleep duration is positively associated with obesity. However, in this study, the focus was on total sleep duration; sleep quality was not explored. Several issues related to sleep among children with ASD have been reported, such as sleep onset and maintenance by parents (60–62). The quality of sleep in children with ASD should be considered in future studies.

Despite this uncertainty in the literature on obesity in children, there is still some preliminary evidence that family structure matters. Family is the primary source of social learning and influence for children (63). It is known that parents, family structure, and family environment can impact the amount of food a child consumes and the level of physical activity of the child (64). Several studies have indicated that two-parent families may be more effective at nurturing children than single-parent families (48, 49). More recent studies have also found associations between family structure (including parent status and number of siblings) and children's physical health outcomes (51, 64–66). Generally, children from single-parent families are more likely than those from two-parent families to be overweight (64). However, in this study, children with ASD from two-parent families had a higher risk of obesity. Family structure considered as an important factor in the family context linked with child development (67). Thus, family structure affects on family functioning and family dynamics (31). Generally married, biological-parent households are providing less stressful family context than other family structures, due to higher levels of economic resources and social support (68). A growing body of the study pointed out that family function and family dynamics such as, family stress, maternal depression, and family connection play a critical role in developing childhood obesity. Since families with children with ASD may have different family functions or dynamics compared to families with typically developed children in terms of the level of stress and interaction among family members, (69) it is important to focus more on family function than family structure to understand how this element could affect on obesity rates of children with ASD. However, there is lack of research examined how the family structure related to obesity in children with ASD, this line of research warrants future attention.

Parents' perceived stress was examined as several studies have indicated that parents of children with ASD tend to have higher stress levels than parents of typically developing children (70–73). Compared to children with other disabilities, children with ASD characterize disabilities in social interaction, impairment of speech and non-verbal communication, homogeneous behavior, and interest, and are unable to communicate with their parents due to late language development (70–73). These behavior patterns of children with ASD may increase parental stress in child rearing. Studies have also reported a higher prevalence of depressive symptoms and lower quality of life in families with children with ASD than in those with children with other developmental disorders such as physical disabilities or chronic health conditions (72–74). Recent data on stress in families of children with ASD have mainly reported stress as perceived by mothers (70, 75–77). The stress level of family members of children with ASD may affect the time and effort invested in fostering a healthy lifestyle because many unhealthy behaviors are associated with increased stress, such as infrequent exercise, alcohol drinking, smoking, sleep disorders, and eating poorly (78). Studies have also indicated that imbalanced dietary patterns and emotional eating are associated with stress (65, 66, 79). Since the family environment affects the amount of food consumed and level of physical activity, the stressful environment of a family with children with ASD may lead to an unhealthy lifestyle that finally affects the obesity level of children with ASD.

The Centers for Disease Control and Prevention guidelines also recommended school- and community-based strategies to prevent childhood obesity by altering the environment and developing relevant policies (80). Since children are nested within families and classes, which are nested within schools, which, in turn, are nested within either school districts or communities, a multilevel approach is needed. By using a clear conceptual framework, the complexity of overweight among children with ASD can be understood systematically and deeply. The long-term goal of this study was to improve the understanding of individual and environmental connections with overweight/obesity mechanisms among children with ASD, which can lead to public health prevention and intervention strategies to reduce overweight/obesity in children with disabilities and ASD. Therefore, comprehensive and multi-sectorial approaches are needed to address the many behavioral and environmental risk factors associated with obesity in children with ASD. A multilevel approach analysis would provide more effective intervention programs and/or policies regarding adolescents' obesity in a multilevel setting (e.g., family, school, district, and state) (81).

This study had the following limitations. First was the use of self-reported measures to assess all variables from the respondents' 1-day recollection, which may be inaccurate because of recall bias, respondent bias, or interview bias. Second, owing to the small sample size (e.g., Hispanic and Asian), attempts to generalize the findings to areas with more diversity must be undertaken with caution. A larger and more diverse sample would yield better results. Third, the dataset excluded households without telephones and/or cellphones, which may result in a biased survey population owing to the underrepresentation of certain segments of the population. Therefore, future studies on obesity prevention in children with ASD should consider these limitations. However, these limitations do not outweigh the contributions of this study.

## Conclusions

This study revealed various multilevel dimensions of how sociological factors are correlated with overweight status in children with ASD. The overweight status of children with ASD was related not only to individual factors but also family and community support. Parental involvement in physical activities and obtaining family and social support is crucial to the overweight status of children with ASD. More attention should be paid to providing children with ASD with family and social support to help them maintain healthy weight. The significance of this study is that it comprehensively explored the obesity trend of children with ASD-related factors to propose multilevel solutions.

## Data availability statement

The original contributions presented in the study are included in the article/supplementary material, further inquiries can be directed to the corresponding author/s.

## Author contributions

TK: conceptualization, methodology, data curation and formal analysis, investigation, writing—original draft preparation, and writing—review and editing. Both authors contributed to the article and approved the submitted version.

## Conflict of interest

The authors declare that the research was conducted in the absence of any commercial or financial relationships that could be construed as a potential conflict of interest.

## Publisher's note

All claims expressed in this article are solely those of the authors and do not necessarily represent those of their affiliated organizations, or those of the publisher, the editors and the reviewers. Any product that may be evaluated in this article, or claim that may be made by its manufacturer, is not guaranteed or endorsed by the publisher.
